# Systemic Oxidative Stress in Women with Ovarian and Pelvic Endometriosis: Role of Hormonal Therapy

**DOI:** 10.3390/jcm11247460

**Published:** 2022-12-16

**Authors:** Anna Biasioli, Anjeza Xholli, Francesca Previtera, Alessandro Balzano, Valentina Capodicasa, Alice Tassi, Ambrogio P. Londero, Angelo Cagnacci

**Affiliations:** 1 Obstetrics and Gynecology Teaching Unit, S. Maria della Misericordia Hospital, 33100 Udine, Italy; 2 Obstetrics and Gynecology Teaching Unit, IRCCS Ospedale San Martino, 16132 Genoa, Italy; 3 Department of Neurology, Rehabilitation, Ophthalmology, Genetics, Maternal and Infant Health (DiNOGMI), University of Genoa, 16132 Genoa, Italy

**Keywords:** endometriosis, oxidative stress, free oxygen radicals, antioxidants, contraception, estrogens, progestins

## Abstract

This study was performed to evaluate the systemic oxidative stress balance in women with either ovarian or deep infiltrating endometriosis (DIE) and any alterations of the same during hormone therapy. Free oxygen radicals (FORT) and free oxidant radical defense (FORD) were measured in the capillary blood of 24 women without endometriosis, 26 women with endometrioma, and 26 women with DIE with or without endometrioma. Endometriosis was diagnosed by clinical and ultrasound assessment. Dietary factors, lifestyle habits, and intake of any substances interfering with the oxidative status were recorded. Women were prescribed contraceptive hormones, and the baseline assessments were repeated at the 3rd month of use, revealing a higher oxidative stress balance (FORT/FORD) in women with endometriosis than in controls (4.75 ± 4.4 vs. 2.79 ± 2.2; *p* = 0.05). The highest values were found in women with DIE (5.34 ± 4.6; *p* = 0.028 vs. controls). Regression analysis revealed an independent link between FORT/FORD and endometrioma (b 2.874, 95% CI 0.345, 5.403; *p* = 0.027) and DIE (b 4.419, 95% CI 1.775, 7.064; *p* = 0.001) but a negative correlation with HDL-cholesterol (b −0.063, 95% CI −0.125, −0.002; *p* = 0.043). In controls, the hormone therapy increased FORT (*p* = 0.003), but also FORD (*p* = 0.012), with the FORT/FORD balance remaining stable (2.72 ± 2.2 vs. 2.73 ± 1.8; *p* = 0.810). In women with endometriosis, FORT remained unchanged, but FORD increased (*p* = 0.004), and the FORT/FORD ratio significantly decreased (4.75 ± 4.4 vs. 2.57 ± 1.76; *p* = 0.002) to values similar to the control levels. These data indicate that systemic oxidative stress balance increased in women with endometriosis, particularly in those with DIE. The hormonal therapy did not change the oxidative stress balance in control women but significantly improved it in women with endometriosis, particularly those suffering from DIE.

## 1. Introduction

Endometriosis is a multifactorial disease defined by the presence of endometrial glands and stroma and associated inflammation in the peritoneal cavity, mainly in the pelvis, but also in other abdominal areas and, on rare occasions, in extra-abdominal sites [1,2,3]. Symptoms are non-specific and include pelvic pain, menstrual pain, and pain upon intercourse. Infertility is one of the main consequences [4].

Retrograde menstruation is considered a key pathogenic mechanism behind the disease, but several additional factors seem to play an important role, including local immunological, inflammatory, angiogenic, and oxidative stress alterations [4]. Oxidative stress is defined as an imbalance between antioxidant defenses—such as catalase, superoxide dismutase, glutathione peroxidase, albumin, ceruloplasmin, vitamin C, vitamin E, uric acid, and HDL-cholesterol—and an excessive production of reactive oxygen species [5]. 

Reactive oxygen species can be evaluated in the blood thanks to the Fenton reaction. This enables the colorimetric assessment of the free oxygen radicals (FORT) liberated by proteins, directly relating them to lipid peroxidation. Antioxidant defenses can also be measured by a colorimetric test (FORD), which assesses their capacity to reduce colored radicals. 

Oxidative stress is involved in the pathological process behind cardiovascular and neurological diseases [5,6]. Published data indicated that it also plays an important role in the pathogenesis and progression of endometriosis [7,8]. Specifically, an overload of oxidizing molecules, such as hemoglobin iron [9,10,11,12], possibly induced by excessive retrograde menstruation, may oversaturate a normal or ineffective peritoneal scavenging system. The peritoneal mesothelium can be injured, favoring the adhesion of ectopic endometrial cells [1,2,13,14] and inducing the amplification of Nf-kB activity [10,15]. This, in turn, promotes disease progression by inducing pro-inflammatory cytokines, growth and angiogenic factors, adhesion molecules, and inducible enzymes [2]. It has been reported that this intraperitoneal condition is accompanied by a more generalized increase in oxidative stress [8]. 

Hormonal contraceptives are recommended for young women with endometriosis due to their simultaneous capacity to treat endometriosis symptoms and prevent pregnancy [16]. In this study, we evaluated the association of systemic oxidative stress with different types of pelvic endometriosis and how the former changes during hormonal contraceptive use.

## 2. Materials and Methods

### 2.1. Ethics Committee

Each woman signed an informed consent for the anonymous use of their data in scientific analysis and publications. The local ethics committee approved the anonymous analysis of the data (No. 1058, approved 28 September 2022). 

### 2.2. Patient Characteristics 

Women were recruited from those attending outpatient services for contraception or endometriosis and pelvic pain at Udine University Hospital between December 2018 and January 2020. Appropriate clinical investigation and counselling were performed by a physician. All women included in the study decided to take an estrogen-based estro–progestin combination pill. Other inclusion criteria were age between 19 and 40 years and regular menstrual cycle length of between 26 and 32 days. Exclusion criteria were the use of any hormonal therapy, nutritional supplements, antioxidants, or vitamins that may interfere with oxidative stress.

### 2.3. Study Design

For each woman, the medical history, weight in kilograms, height in meters, and presence and intensity of pain (menstrual, intermenstrual, and during intercourse) were recorded by a physician. The intensity of each type of pain was quantified by the woman on a 10 cm visual analogue scale (VAS). Pain was considered as present at a VAS score >3. Women were managed in line with standard clinical practice. A detailed history was taken to record factors that may influence oxidative stress. These included: age, current smoking (expressed as the number of cigarettes smoked per day), tea intake (1 unit = 250 mL), physical activity (expressed as the number of minutes of exercise per day), alcohol intake (one unit = 12 g of alcohol), and cocoa consumption (1 unit = 5 g of cocoa). Blood pressure and heart rate were measured 3 times at heart level after 5 min in a sitting position. 

Endometriosis was diagnosed or excluded via clinical assessment and ultrasound investigation. The women were requested to present for a blood testing on a day during the follicular phase of the menstrual cycle (days 4–9). Blood samples were collected at 08:00 after overnight fasting. Therapy was prescribed according to the clinical indications and the women’s request. Records pertaining to women whose prescription was an estradiol-based combined hormone contraceptive were extracted from the database; 33 women received a monophasic combination of micronized estradiol and nomegestrol acetate (13 controls and 20 women with endometriosis), while 43 women received a quadriphasic combination of estradiol valerate and dienogest (11 controls and 32 women with endometriosis). The single-phase combination contained 1.5 mg of estradiol and 2.5 mg of nomegestrol acetate in a 24/4 regimen (Zoely; Theramex Italia, Milan, Italy). The four-phase combination consisted of 2 pills with 3 mg of estradiol valerate, 5 pills with 2 mg of estradiol valerate and 2 mg of dienogest, 17 pills with 2 mg of estradiol valerate and 3 mg of dienogest, 2 pills with 1 mg pf estradiol valerate only, and 2 pills with no hormones (Klaira, Bayer Italia, Milan, Italy). The measurements taken at baseline were repeated after about 3 months of treatment, during the last days of the monthly pill cycle.

### 2.4. Evaluation of the Oxidative Balance 

Factors possibly influencing the oxidative status—ferritin, total cholesterol, HDL-cholesterol, triglycerides, gamma-glutamyl transferase, glucose, and insulin—were measured. All routine blood tests were performed by the same centralized laboratory at Udine University Hospital. The homeostatic model of insulin resistance (HOMA-IR) was calculated using fasting glucose and insulin levels, according to the formula: (glucose mg/dL × insulin (IU/L))/405. 

The oxidative balance was calculated following a colorimetric analysis of free oxygen radicals (FORT) and free oxygen radicals defense (FORD) in finger-tip capillary blood. Specifically, reactive oxygen species were measured in 20 μL of blood via the FORT test (FORM^®^, CR 2000, Callegari, Parma, Italy) [5,6,17,18,19]—a colorimetric assay based on the ability of transition metals, such as iron, to catalyze the breakdown of hydroperoxides (ROOH) into derivative radicals in the Fenton reaction. The radical species interact with an additive (phenylenediamine derivative, 2CrNH_2_), which forms a long-lived, colored, radical cation that can be assayed using a spectrophotometer at 505 nm. The intensity of the color correlates directly with the quantity of radical compounds and, consequently, to the oxidative status of the sample, according to the Beer–Lambert law (Form CR 2000, Callegari). The results are expressed as FORT units, whereby 1 FORT unit corresponds to 0.26 mg/L of H_2_O_2_. The method’s intra-assay and inter-assay coefficients of variation were 3.7% and 6.2%, respectively.

The FORD test provides a measure of the plasma antioxidant system using preformed, stable, colored radicals. It also determines the decrease in absorbance, which is proportional to the blood antioxidant concentration of the sample, according to the Beer–Lambert law. In the presence of an acidic buffer (pH = 5.2) and a suitable oxidant (FeCl3), the chromogen—which contains 4-amino-N, N-diethylaniline sulfate—forms a stable and colored radical cation that is photometrically detectable at 505 nm. Antioxidant compounds in the sample reduce the radical cation of the chromogen, quenching the color and producing a discoloration of the solution in proportion to their concentration. The absorbance values obtained for the samples were compared with a standard curve obtained using Trolox (6-hydroxy-2,5,7,8-tetramethylchroman-2-carboxylic acid)—a permeable cell derivative of vitamin E commonly used as an antioxidant. The intra-assay and inter-assay coefficients of variation were 4.2% and 6.6%, respectively.

### 2.5. Diagnosis of Endometriosis

As indicated by the guidelines (16), the diagnosis of endometriosis was based on the presence of symptoms (pain during menses, between menses, or at intercourse), on a bimanual clinical exam to assess for the presence of stiff nodules, tenderness, and mobility of pelvic organs, and on ultrasound imaging. In some cases, the presence of endometriosis was also confirmed upon laparoscopy.

Ultrasound investigations were performed by an expert, trained practitioner (A.X.) using a Voluson E10 General Electric (GE, General Electric Company, Boston, MA, USA) instrument with a transvaginal probe. The protocol and the terminology reported by the IOTA group were used to identify ovarian endometriosis, mainly characterized by a cyst with ground-glass echogenicity, one to four locules, and no papillation with detectable blood flow [20]. The protocol and the terminology reported by the IDEA group were used to identify the presence of deep infiltrating endometriosis (DIE), assessing the ultrasound findings during the assessment of anterior and posterior compartments [20,21]. Sliding signs of the uterus on the rectum and bladder were sought, and thereafter a systematic approach was used to detect DIE in the urinary bladder, utero-vesical region, ureters, rectovaginal septum, vaginal wall, rectovaginal nodules, uterosacral ligaments, rectum, rectosigmoid junction, and sigma [20,21]

Women without clinical and ultrasonographic signs of endometriosis were categorized as controls. Women with only ovarian endometriosis constituted the Endometrioma group. Women with a diagnosis of DIE, with or without associated endometrioma, were allocated to the DIE group. 

### 2.6. Study Power 

The study was powered for a difference between the two groups equal to or greater than the mean of the SD of the two groups. By setting the type I error at 0.05 and the type II error at 0.2, 23 subjects in each group would be sufficient to document a between-group difference. A sample size of 18 would be sufficient to document a within-group difference with an SD deviation of the difference equal to 1.5 times the value of the difference. 

### 2.7. Statistical Analysis

Comparison between the two groups was performed via the Student’s *t*-test, and within group-comparison by the paired *t*-test. When the data were not normally distributed, the Mann–Whitney rank sum test and the Wilcoxon signed-rank test were used for between-group and within-group comparisons, respectively. Comparison among multiple groups was performed by analysis of variance followed by Fisher’s post-hoc test. Simple regression analysis was used to correlate FORT, FORD, or FORT/FORD with determinants such as age, BMI (kg/m^2^), physiological and nutritional aspects (smoking, physical activity, tea intake, alcohol intake, cocoa intake), and biochemical parameters (ferritin, total cholesterol, HDL-cholesterol, triglycerides, gamma-glutamyl transferase, glucose, insulin). Categorical data, namely, the presence of endometrioma (y/n) or the presence of DIE (y/n), were entered as dummy variables, with the absence of those diseases considered as the reference group. To identify factors independently related to oxidative stress, only factors individually related to FORT, FORD, or FORT/FORD were entered into the multiple regression analysis. Endometrioma and DIE were forced into the analysis even if, in the simple regression analysis, they were not correlated to the indices of oxidative stress.

All analyses were performed using the statistical software package StatView 5.01 (SAS Institute Inc., Cary, NC, USA). All results are expressed as means and standard deviations. A *p* value < 0.05 was considered statistically significant.

## 3. Results

### 3.1. Clinical Characteristics

Among the 78 patients initially included, a complete set of data was only available for 76 women, of whom 52 had a diagnosis of endometriosis (cases), and 24 were asymptomatic and had no ultrasound sign of endometriosis (controls). Among the cases, 26 had clinical and ultrasound features of isolated ovarian endometriosis, and 26 of DIE, with or without ovarian endometriosis. 

The general characteristics of the enrolled women are reported in Table 1. The mean age of the controls and of women with endometriosis was 33.1 ± 6.4 yrs. and 33.6 ± 6.2 yrs. (*p* = 0.745), and their respective BMI values were 23.4 ± 4.5 and 22.8 ± 4.1 (*p* = 0.585).

Women with endometriosis showed a higher daily intake of alcohol (0.163 ± 0.322 vs. 0.013 ± 0.04 units/day; *p* = 0.033) and a lower intake of cocoa (0.877 ± 0.88 vs. 1.59 ± 1.66 units/day, *p* = 0.039). These differences, along with a lower tea intake (0.20 ± 0.37 vs. 0.67 ± 0.88 units/day; *p* = 0.017), were significant only in women with DIE, while they did not reach statistical significance in women in the Endometrioma group (Table 1). 

Higher levels of blood glucose (88.4 ± 10.7 vs. 82.1 ± 8.11 mg/dL; *p* = 0.026), lower levels of triglycerides (60.0 ± 22.9 vs. 85.2 ± 59.4 mg/dL; *p* = 0.013), and not significantly lower (*p* = 0.06) levels of HDL-cholesterol were observed in women with endometriosis (Table 2).

### 3.2. Pretreatment Analyses

#### Oxidative Stress

Compared to the controls, women with endometriosis showed no significantly higher values of FORT and no significantly lower values of FORD. However, the FORT/FORD ratio was significantly higher in women with than in those without endometriosis (4.75 ± 4.4 vs. 2.79 ± 2.2; *p* = 0.050) (Table 1). 

Women with only endometrioma had FORT, FORD, and FORT/FORD values similar to those of the controls. However, women with DIE, although not exhibiting significantly higher levels of FORT (2.25 ± 1.17 vs. 1.75 ± 0.99; *p* = 0.096), had significantly lower levels of FORD (0.55 ± 0.26 vs. 0.80 ± 0.35; *p* = 0.043) and therefore a significantly higher FORT/FORD ratio (5.34 ± 4.6 vs. 2.79 ± 2.2; *p* = 0.028) (Table 2).

### 3.3. Regression Models

#### 3.3.1. FORT

Upon simple linear regression analysis, FORT was correlated positively with BMI (*p* = 0.044) and physical activity (*p* = 0.016) and negatively with HDL-cholesterol (*p* = 0.037) (Table 2). No correlation was observed with the presence of endometrioma or DIE. However, in conditioned multiple linear regression analysis, FORT was correlated positively only with DIE (b 0.841 95%CI 0.284, 1.398; *p* = 0.004) and negatively with HDL-cholesterol (b −0.025 95%CI −0.040, −0.011; *p* = 0.001) (Table 3).

#### 3.3.2. FORD

Upon simple linear regression analysis, FORD was correlated positively with tea intake (*p* < 0.036) and negatively with DIE (*p* = 0.008), glucose (*p* = 0.001) and ferritin (*p* = 0.012) (Table 3). Upon multiple regression analysis, FORD remained negatively correlated with glucose alone (b −0.017 95%CI −0.027, −0.007; *p* = 0.001) (Table 3).

#### 3.3.3. FORT/FORD

Upon simple regression analysis, the FORT/FORD ratio was positively correlated with glucose (*p* = 0.035) and triglycerides (*p* = 0.015) and negatively with HDL-cholesterol (*p* = 0.039) (Table 2). Upon conditioned multiple regression analysis, the FORT/FORD ratio was positively correlated with the presence of both endometrioma (b 2.874 95% CI 0.345, 5.403; *p* = 0.027) and DIE (b 4.419 95% CI 1.775, 7.064; *p* = 0.001) and negatively correlated with HDL-cholesterol (b −0.063 95% CI −0.125, −0.002; *p* = 0.043) (Table 3).

### 3.4. Effect of Hormonal Contraceptives

**Figure 1:**
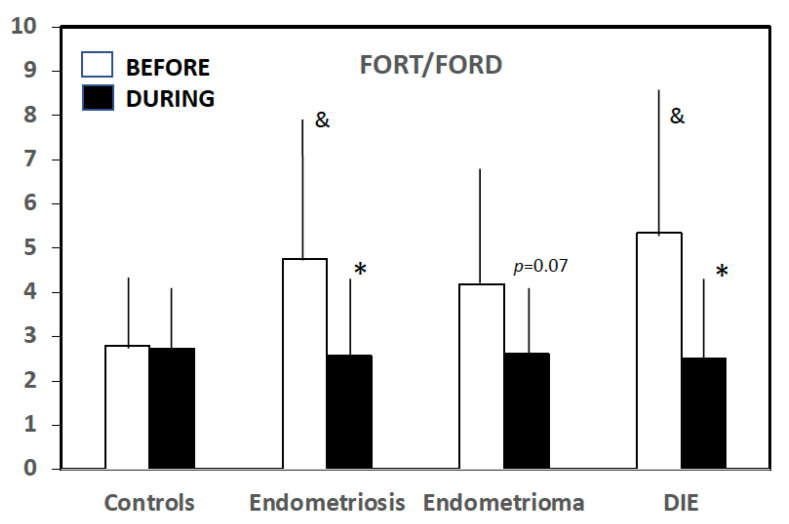
FORT/FORD values before and during hormonal contraception. * Significant vs. pretreatment values. ^&^ Significant vs. controls (please, see text for details).

In control women, hormonal contraceptives increased both FORT (0.98 ± 0.93; *p* = 0.003) and FORD (0.416 ± 0.63; *p* = 0.012), and the FORT/FORD ratio remained stable (0.07 ± 1.34; *p* = 0.810) (Table 3) (Figure 1). In women with endometriosis, however, FORT remained stable (−0.15 ± 1.4; *p* = 0.457), while FORD increased (0.311 ± 0.72; *p* = 0.004), leading to a significant decrease in the FORT/FORD ratio (−2.24 ± 4.8; *p* = 0.002). This effect was significant for women with DIE (−2.80 ± 4.9; *p* = 0.011) and close to significance in the Endometrioma group (−1.75 ± 4.6; *p* = 0.068) (Table 4) (Figure 1).

## 4. Discussion

The data presented indicated that women with endometriosis, particularly with DIE, had increased systemic oxidative stress, as assessed by the ratio of free oxygen radicals (FORT) to antioxidants (FORD). The greater oxidative stress seen in these women was, however, improved by the administration of estradiol-based hormonal contraceptives. 

Alterations in systemic oxidative stress have been assessed by FORT and FORD, to indicate overall systemic free oxygen radicals and antioxidant defenses, respectively, in other clinical conditions [5,6,17,18]. The FORT and FORD levels appeared as appropriate means of investigating the oxidative balance in this sample, showing that oxidative stress was lower when the antioxidant defenses were higher, as in the case of higher levels of HDL-cholesterol, which bears on its surface the antioxidant enzyme paraxonase [19,22], or with a higher intake of antioxidants, such as the phenolic compounds contained in tea [23] or cocoa [24]. Similarly, the FORT/FORD oxidative stress balance was increased in all those conditions associated with a higher production of free radicals, namely, obesity [19,22], glucose metabolism alterations [5,25], increased levels of ferritin [26], alcohol intake [27], and physical activity [28]. The FORT/FORD ratio was also higher in women with DIE than in controls, indicating that the increased intraperitoneal oxidative stress was reflected in the systemic circulation [29]. 

A relationship between oxidative status and the severity of endometriosis was previously investigated, albeit with contrasting results [30]. Either no correlation [31,32] or higher lipid peroxidation in the peritoneal fluid [33] and serum [33,34] and lower superoxide dismutase in the peritoneal fluid were observed in the case of severe DIE [33]. In our study, the oxidative status was more closely associated with DIE than with endometrioma alone. Indeed, DIE nodules are associated with reduced vascularization, and the consequent ischemia may increase oxidative stress, [35]. That being said, when corrected for confounding variables, women with endometrioma alone also had a higher FORT/FORD ratio than the controls. DIE is associated with a reduced capability of spontaneous conception, and endometrioma with a reduced oocyte quality from the same ovary [16]. These data showing an increased oxidative stress balance in any type of endometriosis support the association between the presence of the disease and infertility [16].

Cross-sectional studies evaluating antioxidants (vitamin C, vitamin E, thiol products, selenium, copper, zinc) and oxidized LDL reported an increased oxidative stress in users of contraceptives containing ethynyl estradiol (EE) and different progestins [36,37]. An increase in total reactive oxygen products (FORT), with no evaluation of FORD, was also observed in users vs. non-users of different contraceptives, mainly containing EE [38]. Similarly, in two prospective studies not evaluating blood antioxidant defenses, the total reactive oxygen products were increased by the administration of a pill containing 50 mcg of EE and levonorgestrel [39,40]. In our control women, FORT increased during hormone contraception, but the FORT/FORD ratio remained stable as there was a concomitant increase in FORD. This is at variance with cross-sectional [38] and prospective studies [39,40] that did not evaluate the antioxidant defenses or with cross-sectional studies that did not evaluate the total antioxidant defense. The ongoing use of estradiol vs. ethynyl estradiol may also have made the difference, as ethynyl estradiol has a stronger metabolic impact on the liver and is transformed into catechol estrogens—factors possibly leading to an increase in oxidative stress [37,39]. 

It is interesting to observe the effect of hormonal contraceptives on the oxidative stress of women with an already elevated oxidative stress balance, i.e., those with pelvic endometriosis. In these women, FORT decreased, while FORD significantly increased. Whereas before treatment, the FORT/FORD ratio was markedly higher than in controls, during estrogen-based hormonal therapy, the FORT/FORD ratio of women with endometriosis returned to the levels seen in control women. 

An increased oxidative status seems to be involved in pain development and disease progression. In the present study, we did not grade the pain symptoms during treatment, but many studies have indicated hormonal contraceptives as a valuable tool for treating symptoms of endometriosis and even for reducing its post-surgical recurrence [16]. Therapies aimed at improving the oxidative status are considered a way to treat endometriosis and its associated symptoms [29,30,37,38,39,40,41,42,43], and the data presented here indicate that amelioration of the oxidative stress balance may represent an additional benefit of the use of hormonal contraceptives in the long-term management of women with endometriosis.

### Strengths and Limitations

The number of women tested in this study was higher than in most of the studies published to date [30]. Furthermore, endometriosis was diagnosed by clinical and ultrasound signs and through surgery, in line with guidelines indicating that the disease should be diagnosed based on symptoms, clinical examination, and ultrasonography [16]. Systematic approaches to the identification of ovarian and pelvic endometriosis by transvaginal sonography have been published [20,21]. The procedure is considered highly reliable in the hand of expert sonographers. In our case, the procedure was performed by the same sonographer, with specific expertise in identifying ovarian and pelvic endometriosis (A.X.). 

However, we did not look for alterations in selected specific oxidant and antioxidant enzymes and substances, but rather considered oxidative stress balance as a whole. Moreover, the study was performed in a single center on white women only. Other studies in different settings and on a more inclusive population are required.

## 5. Conclusions

Measuring FORT and FORD in capillary blood may be an easy means of testing the oxidative status in women with endometriosis and of monitoring its alteration during hormone contraceptive therapy. Estradiol-based hormonal contraceptives may reduce oxidative stress in women with endometriosis.

## Figures and Tables

**Table 1:** Mean and standard deviation (mean ± SD) of general and lifestyle characteristics observed in controls (*n* = 24) and cases (*n* = 52) considered together and divided by type of endometriosis, i.e., either endometrioma alone (*n* = 26) or deep pelvic infiltrating endometriosis (DIE) with or without endometrioma (*n* = 26). Significance of the between-group comparisons is reported.

	A Controls	B Endometriosis	B vs. A *p* Value	C Endometrioma	C vs. A *p* Value	D DIE	D vs. A *p* Value	D vs. C *p* Value
Age (yrs.)	33.1 ± 6.4	33.6 ± 6.2	0.745	33.9 ± 6.5	0.674	33.4 ± 5.9	0.768	0.898
BMI (kg/m^2^)	23.4 ± 4.5	22.8 ± 4.1	0.585	23.27 ± 4.23	0.917	22.29 ± 4.11	0.383	0.421
Smoking (n/day)	1.09 ± 2.9	2.15 ± 4.5	0.315	2.64 ± 5.45	0.192	1.58 ± 3.16	0.687	0.360
Tea (units/day)	0.67 ± 0.88	0.36 ± 0.55	0.07	0.50 ± 0.63	0.360	0.20 ± 0.37	0.017	0.103
Activity (min/day)	4.54 ± 9.86	10.46 ± 19.5	0.182	10.1 ± 20.7	0.262	10.8 ± 18.5	0.224	0.886
Alcohol (unit/day)	0.013 ± 0.04	0.163 ± 0.32	**0.033**	0.123 ± 0.264	0.158	0.210 ± 0.38	0.016	0.252
Cocoa (unit/die)	1.51 ± 1.66	0.877 ± 0.88	**0.039**	1.09 ± 0.79	0.214	0.625 ± 0.92	0.012	0.143

DIE: Deep Infiltarting Endometriosis; Bold values—significant values (*p* value < 0.05).

**Table 2:** Mean and standard deviation (mean ± SD) of general health characteristics and blood test results observed in controls (*n* = 24) and cases (*n* = 52) considered together or divided by type of endometriosis, i.e., either endometrioma alone (*n* = 26) or deep pelvic infiltrating endometriosis (DIE) with or without endometrioma (*n* = 26). Significance of the between-group comparisons is reported.

	A Controls	B Endometriosis	B vs. A *p* Value	C Endometrioma	C vs. A *p* Value	D DIE	D vs. A *p* Value	D vs. C *p* Value
BP sys (mmHg)	113.2 ± 10.0	113.4 ± 12.6	0.927	113.2 ± 9.9	0.992	113.7 ± 15.4	0.873	0.873
Bp dia (mmHg)	71.82 ± 9.8	74.23 ± 10.1	0.349	74.6 ± 9.7	0.331	73.7 ± 10.8	0.520	0.752
HR (beats/min)	77.2 ± 12.2	72.6 ± 10.2	0.099	72.57 ± 8.99	0.142	72.58 ± 11.6	0.157	0.997
Glucose (mg/dL)	82.1 ± 8.11	88.4 ± 10.7	**0.026**	89.1 ± 12.4	**0.039**	87.3 ± 8.7	0.076	0.687
Insulin (UI/L)	14.1 ± 11.1	9.60 ± 8.87	0.130	8.49 ± 8.42	0.058	10.8 ± 9.37	0.269	0.391
HOMA-IR index	2.97 ± 2.47	2.18 ± 2.17	0.206	2.00 ± 2.26	0.170	2.36 ± 2.1	0.394	0.579
Tot. Chol. (mg/dL)	160.1 ± 31.5	170.7 ± 29.1	0.197	162.8 ± 30.1	0.760	179.3 ± 25.9	**0.038**	**0.049**
HDL-Chol. (mg/dL)	60.4 ± 16.7	68.8 ± 15.7	0.060	66.5 ± 14.0	0.218	71.3 ± 17.4	**0.032**	0.293
HDL/Tot. Chol.	0.38 ± 0.09	0.40 ± 0.07	0.335	0.41 ± 0.07	0.238	0.39 ± 0.07	0.613	0.461
Triglyc. (mg/dL)	85.2 ± 59.4	60.0 ± 22.9	**0.013**	60.1 ± 22.9	**0.028**	59.9 ± 23.2	**0.029**	0.981
Ferritin (mcg/L)	38.9 ± 29.5	28.0 ± 25.4	0.138	27.7 ± 32.9	0.151	29.0 ± 13.8	0.239	0.791
FORT mM/L H_2_O_2_	1.75 ± 0.99	2.11 ± 1.03	0.167	1.98 ± 0.908	0.419	2.25 ± 1.17	0.096	0.342
FORD mM/L Trolox	0.80 ± 0.35	0.71 ± 0.47	0.404	0.86 ± 0.57	0.658	0.55 ± 0.26	**0.043**	**0.011**
FORT/FORD	2.79 ± 2.2	4.75 ± 4.4	0.050	4.19 ± 4.15	0.214	5.34 ± 4.6	**0.028**	0.298

Bold values—significant values (*p* value < 0.05).

**Table 3:** Simple linear regression analysis and multiple linear regression analysis of factors significantly correlated with blood oxidant (FORT) and antioxidant activity (FORT) and FORT/FORD ratio in the examined women, of which 24 were without endometriosis, 26 were with endometrioma, and 26 were with DIE.

	Simple Regression b (95% CI)	R2; *p*	Multiple Regression b (95% CI)	*p*
FORT			R2 = 0.388; 0.0001	
Endometrioma (y/n)	−0.029 (−0.524, 0.467)	0.0001; 0.907	0.434 (−0.141, 1.008)	0.136
DIE (y/n)	0.377 (−0.219, 0.882)	0.030; 0.142	0.841 (0.284, 1.398)	**0.004**
BMI (Kg/m^2^)	0.058 (0.02, 0.115)	0.06; **0.044**	0.037 (−0.028, 0.102)	0.256
Activity (min/day)	0.017 (0.003, 0.030)	0.078; **0.016**	0.130 (−0.003, 0.028)	0.116
HDL-cholest. (mg/dL)	−0.015 (−0.029, −0.001)	0.067; **0.037**	−0.025 (−0.040, −0.011)	**0.001**
FORD			R2 = 0.310; 0.0004	
Endometrioma (y/n)	0.189 (−0.022, 0.400)	0.044; 0.078	0.166 (−0.100, 0.432)	0.216
DIE (y/n)	−0.286 (−0.495, −0.08)	0.084; **0.008**	−0.062 (−0.319, 0.195)	0.630
Tea (units/day)	0.159 (0.011, 0.308)	0.048; **0.036**	0.089 (−0.052, 0.231)	0.213
Glucose (mg/dL)	−0.017 (−0.027, −0.007)	0.137; **0.001**	−0.017 (−0.027, −0.007)	**0.001**
Ferritin (mcg/L)	−0.007(−0.012, −0.002)	0.095; **0.012**	−0.004 (−0.009, 0.001)	0.127
FORT/FORD			R2 0.332; 0.0006	
Endometrioma (y/n)	0.074 (−1.865, 2.013)	0.0009; 0.939	2.874 (0.345, 5.403)	**0.027**
DIE (y/n)	1.785 (−0.145, 3.714)	0.035; 0.069	4.419 (1.775, 7.064)	**0.001**
Glucose (mg/dL)	0.108 (0.008, 0.208)	0.068; **0.035**	0.058 (−0.039, 0.155)	0.237
HDL-cholest. (mg/dL)	−0.064 (−0.124, −0.003)	0.065; **0.039**	−0.063 (−0.125, −0.002)	**0.043**
Triglycerides (mg/dL)	0.032 (0.006, 0.057)	0.075; **0.015**	0.024 (−0.004, 0.052)	0.097

DIE: Deep Infiltarting Endometriosis; Bold values—significant values (*p* value < 0.05).

**Table 4:** Mean and standard deviation (mean ± SD) of blood oxidant (FORT), antioxidant (FORD), and the oxidant/antioxidant ratio (FORT/FORD) observed prior to and after 3 months of estradiol-based hormonal contraception in women without (controls; *n* = 24) and with endometriosis (*n* = 52), of which 26 had endometrioma alone, and 26 had deep infiltrating endometriosis, associated or not with ovarian endometriosis.

	Before	During	Net	*p*
FORT mM/L H_2_O_2_				
Control	1.75 ± 0.93	2.4 ± 0.74	0.98 ± 0.93	**0.003**
Endometriosis	2.11 ± 1.03	1.96 ± 1.08	−0.15 ± 1.4	0.457
Endometrioma	1.98 ± 0.91	1.68 ± 0.71	−0.304 ± 0.63	0.230
DIE	2.25 ± 1.17	2.29 ± 1.25	0.03 ± 1.58	0.920
FORD mM/L Trolox				
Control	0.80 ± 0.34	1.20 ± 0.62	0.416 ± 0.63	**0.012**
Endometriosis	0.70 ± 0.47	1.06 ± 1.08	0.311 ± 0.72	**0.004**
Endometrioma	0.86 ± 0.57	0.90 ± 0.53	0.06 ± 0.76	0.711
DIE	0.55 ± 0.26	1.14 ± 0.51	0.58 ± 0.56	**0.001**
FORT/FORD				
Control	2.79 ± 2.2	2.73 ± 1.8	0.07 ± 1.34	0.810
Endometriosis	4.75 ± 4.4	2.57 ± 1.76	−2.24 ± 4.8	**0.002**
Endometrioma	4.19 ± 4.1	2.61 ± 1.83	−1.75 ± 4.6	0.068
DIE	5.34 ± 4.6	2.50 ± 1.71	−2.80 ± 4.9	**0.011**

DIE: Deep Infiltarting Endometriosis; Bold values—significant values (*p* value < 0.05).

## Data Availability

The data that support the findings of this study are available, but restrictions apply to the divulgation of these data, which were used under license for the current study and so are not publicly available. Data are however available from the authors upon reasonable request and with permission of the Ethics Committee.
